# Supplementary material for: Genome editing in plants using the compact editor CasΦ
Source: Proc Natl Acad Sci U S A. 2023 Jan 18;120(4):e2216822120. doi: 10.1073/pnas.2216822120 (PMC9942878; doi:10.1073/pnas.2216822120)
Supplement: Supplementary file 1 — Appendix 01 (PDF) [file pnas.2216822120.sapp.pdf]

PNAS

Supporting Information for
Genome editing in plants using the compact editor CasΦ.

Zheng Li [a], Zhenhui Zhong [a], Zhongshou Wu [a], Patrick Pausch [b], Basem Al-Shayeb [c,d], Jasmine Amerasekera[e], Jennifer A. Doudna [c,f,g,h,i,j,k] and Steven E. Jacobsen [a,l,1]

[1] To whom correspondence may be addressed. Email: jacobsen@ucla.edu

This PDF file includes:

Figures S1 to S9
Tables S1 to S7

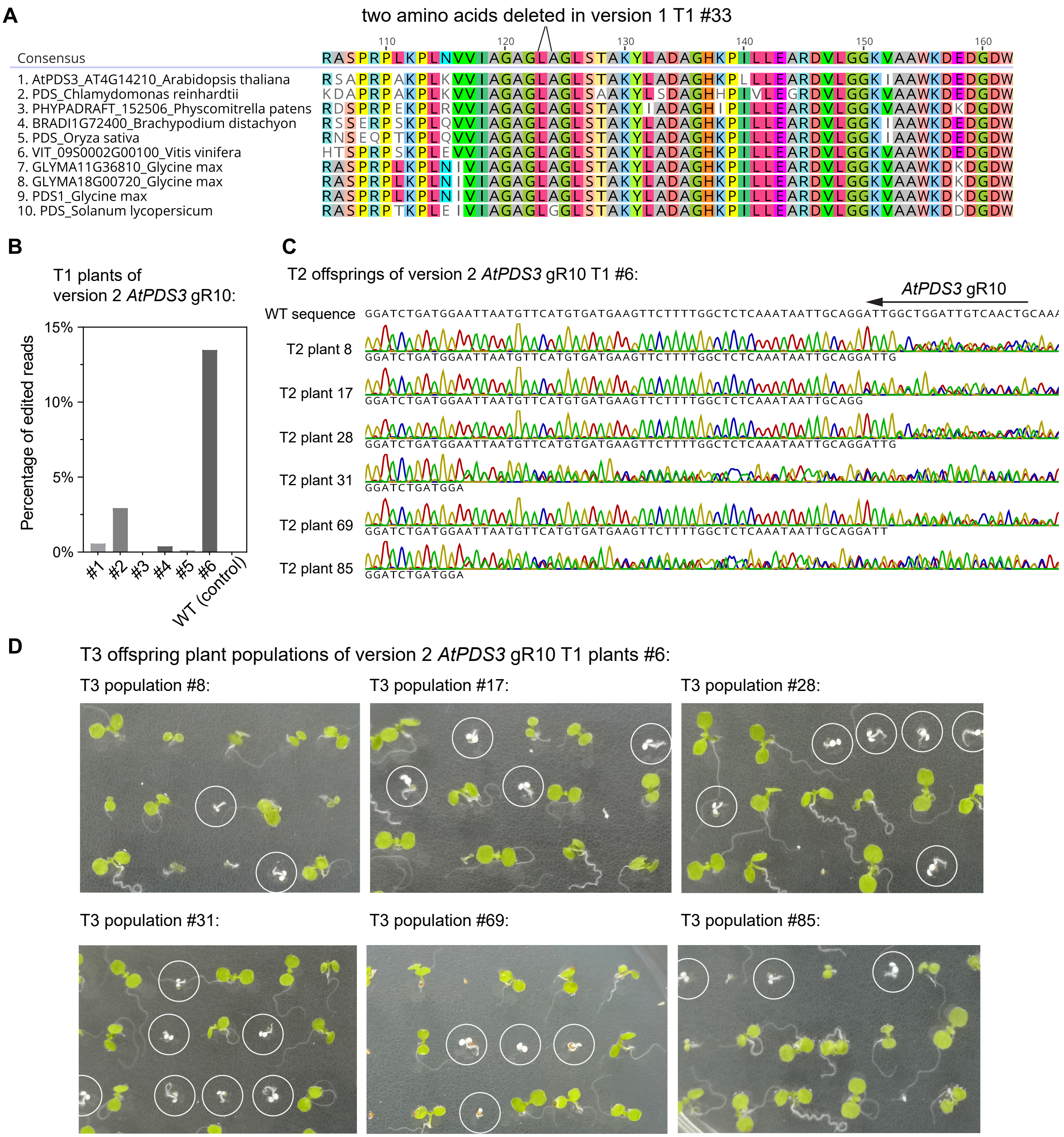

Fig. S1. CasΦ-2 mediated editing of the *AtPDS3* gene with version 1 and version 2 constructs. (A) AtPDS3 homolog protein sequences from different species were aligned with Clustal Omega by the Geneious software, with the two amino acids deleted in version 1 construct T1 plant #33 labeled. (B) Amplicon sequencing results of T1 plant leaves of version 2 *AtPDS3* gRNA10 construct. (C) Sanger sequencing results of the *AtPDS3* gRNA10 target region of six out of 96 total seedlings from the T2 population of version 2 *AtPDS3* gR10 T1 plant #6, showing that they are heterozygous for mutation in this region. (D) Seedlings of six T3 offspring plant populations of version 2 *AtPDS3* gRNA10 T1 plant #6, corresponding to the six T2 plant indicated in (C), with albino seedlings circled.

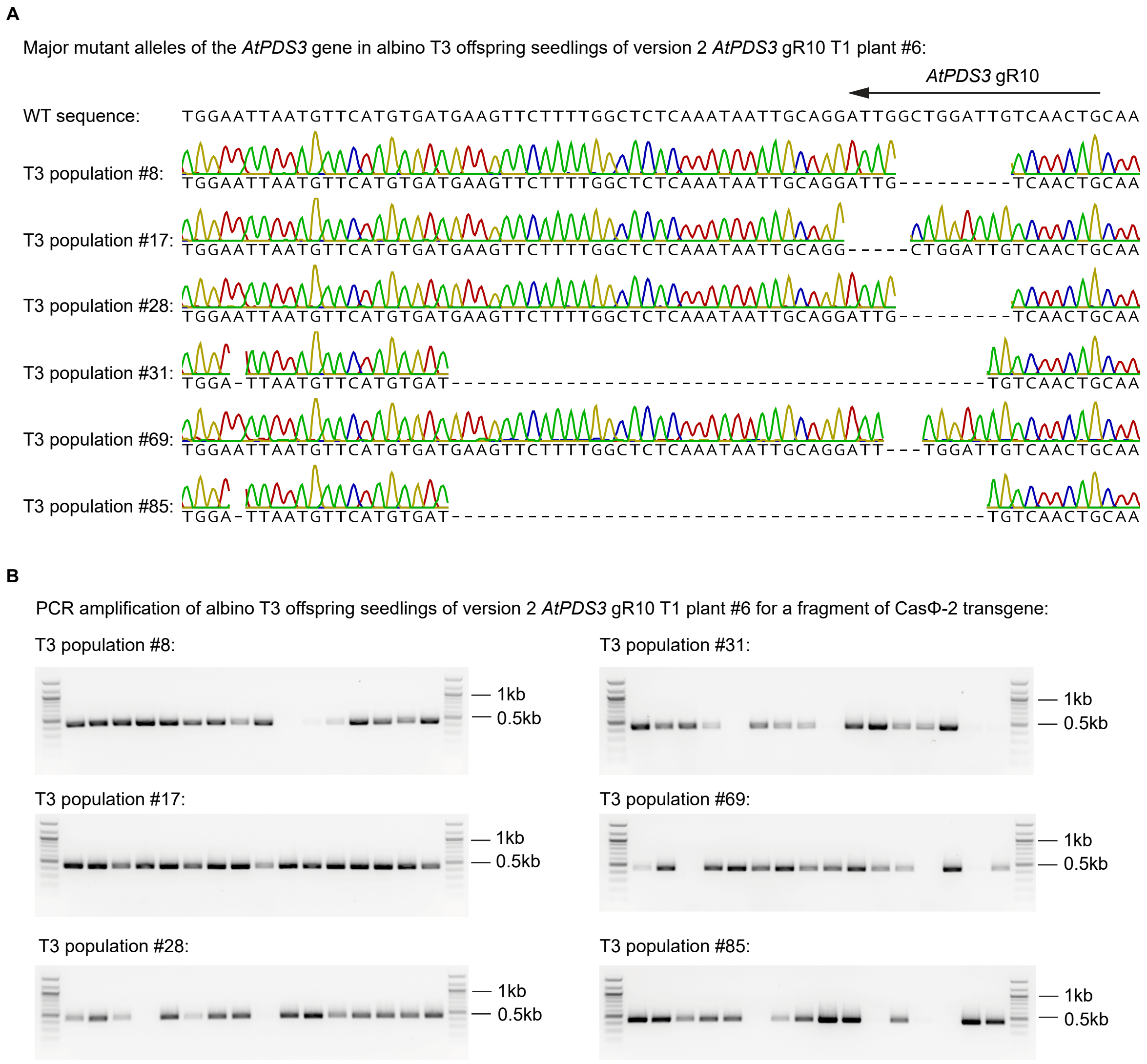

Fig. S2. Characterization of the editing of the *AtPDS3* gene by CasΦ-2 with the version 2 construct in the T3 generation. (A) multiple albino seedlings from T3 populations corresponding to the T2 plants in Fig. S1*C* were sanger sequenced and the major mutant alleles are displayed. (B) PCR amplification of the DNA of 16 randomly selected albino seedlings from the T3 populations in (A) for a fragment of the CasΦ-2 transgene. Successful DNA extraction was suggested by successful PCR amplification of the *AtPDS3* gRNA10 targeted region followed by sanger sequencing with the same DNA samples from these 16 albino seedlings from each of the T3 populations, with major mutation pattern as shown in (A).

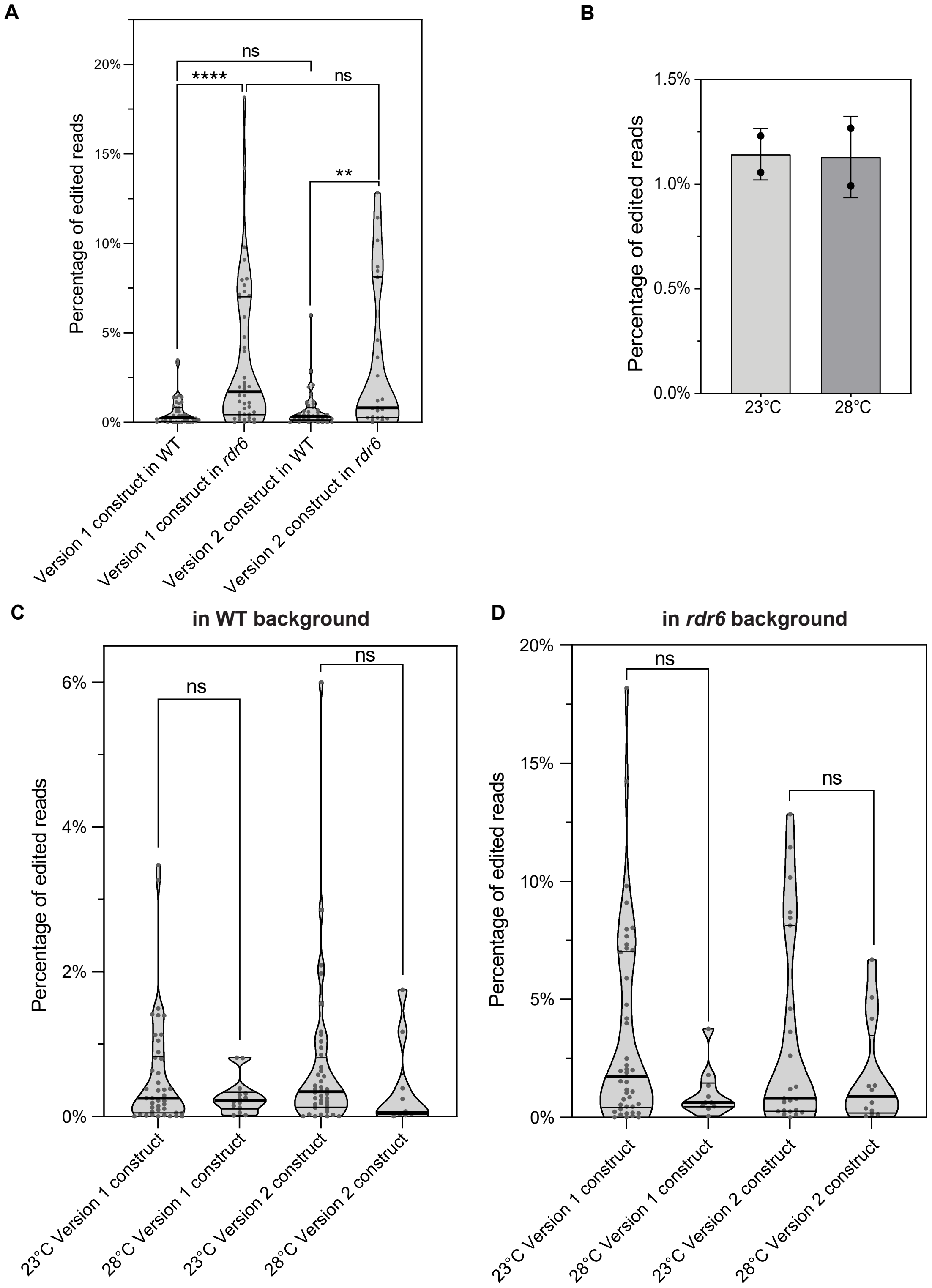

Fig. S3. The impact of transgene silencing and temperature on target gene editing efficiency by CasΦ-2. (A) Version 1 and version 2 constructs with *AtPDS3* gRNA10 were transformed into Col-0 (WT) and *rdr6-15* (*rdr6*) backgrounds. Editing efficiencies in T1 leaves determined by amplicon sequencing were plotted. The numbers of independent T1 plants (n) scored for each population are: n=43 for version 1 construct in WT background, n=42 for version 1 construct in *rdr6* background, n=41 for version 2 construct in WT background and n=23 for version 2 construct in *rdr6* background. (B) The Col-0 protoplasts were transfected with version 2 construct with *AtPDS3* gRNA10 and incubated at 23°C and 28°C. Two replicate transfections

were performed for each temperature and the editing efficiency of each individual transfection, as well as the mean and standard deviation of the two replicates were plotted. (C) and (D), version 1 and version 2 constructs with *AtPDS3* gRNA10 were transformed into WT background (C) and the *rdr6* mutant background (D). T1 plants were incubated constantly at 23°C (23°C sets) or initially at 28°C for 2 weeks then at 23°C (28°C sets). Editing efficiencies in T1 leaves were plotted. The numbers of independent T1 plants (n) scored for each population are: in the WT background (C), n=43 for the set of 23°C version 1 construct, n=15 for the set of 28°C version 1 construct, n=41 for the set of 23°C version 2 construct and n=10 for the set of 28°C version 2 construct; In the *rdr6* background (D), n=42 for the set of 23°C version 1 construct, n=10 for the set of 28°C version 1 construct, n=23 for the set of 23°C version 2 construct and n=12 for the set of 28°C version 2 construct. In (A), (C) and (D), truncated violin plots and all data points are shown, with median and quartiles indicated (thicker and thinner line, respectively). One or zero outliers were removed from each population as determined by the Grubbs method (Alpha =0.0001) for clearer viewing of the major population. Mann-Whitney test was used to calculated the P value for each comparison indicated. ns, non-significant, P>0.05; **, 0.01<P<0.001; ****, P<0.0001.

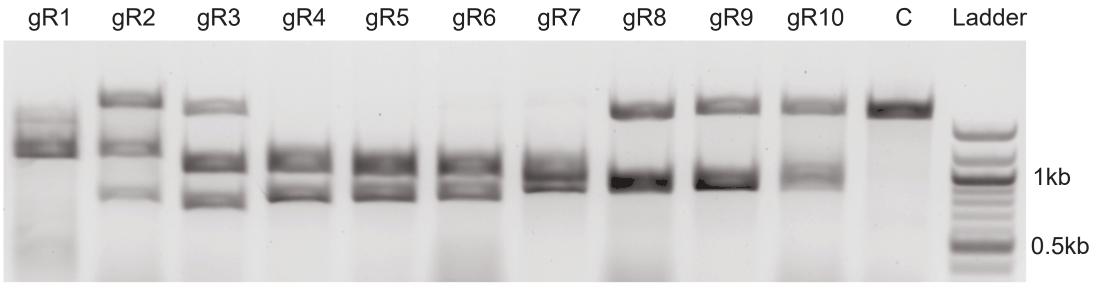

Fig. S4. *In vitro* cleavage of PCR amplified *FWA* gene fragment by CasΦ-2 RNP with *FWA* gRNA1 to gRNA10. A1.57kb *FWA* gene fragment spanning all gRNA target regions was amplified by PCR and gel purified. The *FWA* gene fragment was incubated with CasΦ-2 RNPs containing gRNA1 to gRNA10 (gR1 to gR10) and a scrambled gRNA control (C) at 37°C for 1 hour. Reactions were stopped by adding EDTA and digestion of CasΦ-2 protein with proteinase K. 2% agarose gels were used to visualize the cleavage products along with a DNA ladder for sizing.

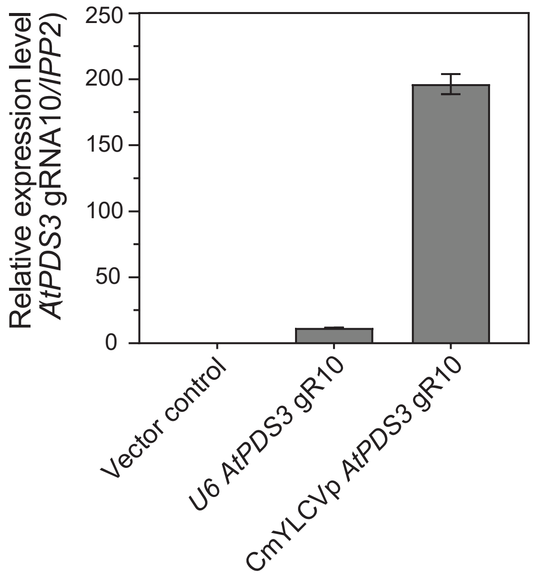

Fig. S5. Comparison of the level of the *AtPDS3* gRNA10 driven by the U6 and the CmYLCV promoter in protoplasts. Real-time quantitative PCR was used to measure the level of *AtPDS3* gRNA10 expression level in protoplasts transfected with the same amounts of the version 2 U6::*AtPDS3* gRNA10 plasmid and the version 2 CmYLCVp::*AtPDS3* gRNA10 plasmid. Protoplasts transfected with the version 2 U6::*AtPDS3* gRNA8 plasmid was used as the vector control to evaluate basal noise level of the primer pair used for the *AtPDS3* gRNA10 amplification. The *IPP2* gene was used as a reference gene for normalization. Three technical replicates were performed. Mean and standard error of the relative quantity calculated by the Bio-Rad CFX software are plotted.

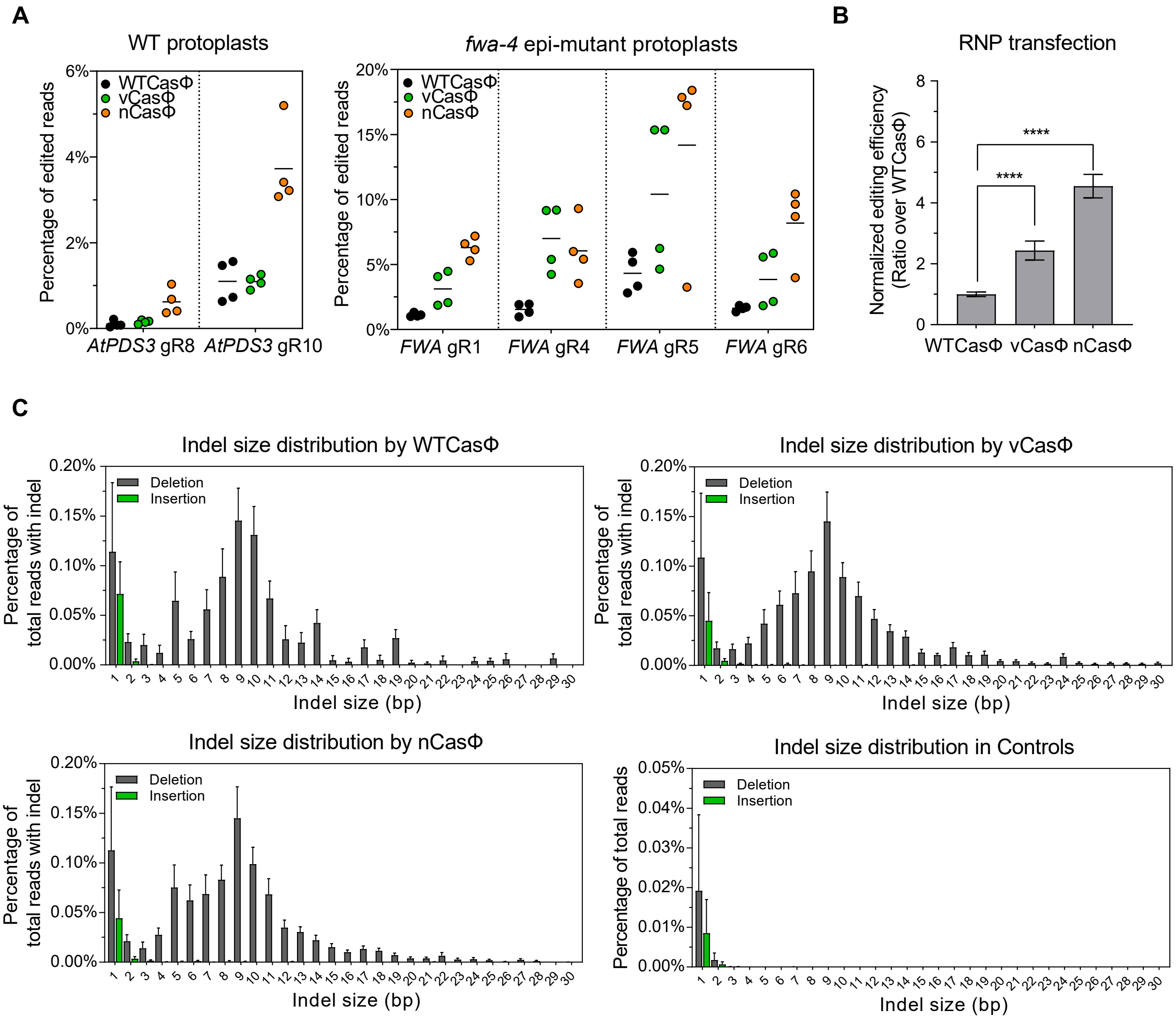

Fig. S6. Comparison of editing efficiency and indel size distribution profile by the vCasΦ and nCasΦ variants and WTCasΦ in protoplasts. (A) RNPs reconstituted with WTCasΦ, vCasΦ and nCasΦ proteins and guide RNAs as indicated were transfected into protoplasts prepared from Col-0 plants (WT) (left panel) and from *fwa-4* epi-mutant plants (right panel). Individual replicate values and mean of the four replicates of each test were plotted. (B) Target gene editing efficiencies in (A) were normalized by calculating the ratio of editing efficiencies over that of mean editing efficiency by WTCasΦ for each guide RNA. Mean and standard error of the normalized editing efficiencies for all gRNAs were plotted. Unpaired t-test was used to calculate P value of indicated comparisons. ****, $P<0.0001$. (C) The indel size distribution was calculated as the percentage of reads of a particular insertion or deletion size, from 1 bp to 30 bp, among all reads with indels for each protoplast transfections in Fig. 4*A*. Mean and standard error of the indel size distributions of all guide RNAs in Fig. 4*A* are plotted. For the control samples (bottom right panel), protoplasts transfected with the HBT-sGFP plasmid were amplified for the target regions of the six guide RNAs used in Fig. 4*A* (six amplicon sequencing for the control samples in total). The indel size distribution was calculated as the percentage of reads of a particular insertion or deletion size among all reads.

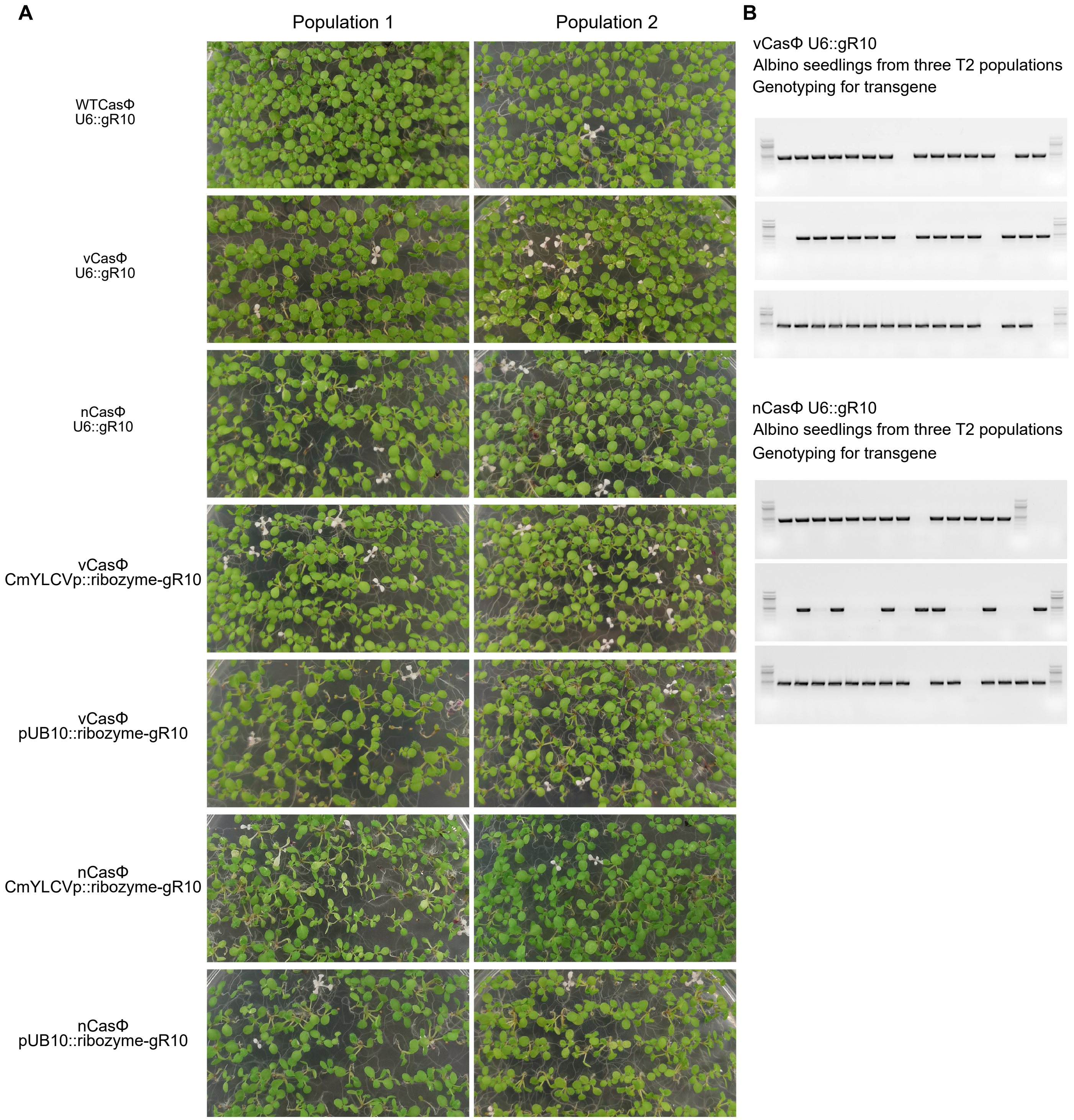

Fig. S7. Heritability analysis of the editing of the *AtPDS3* gene by the vCasΦ and nCasΦ variants in the T2 generation. (A) Pictures of two representative T2 populations of transgenic plants of indicated constructs in the *rdr6-15* background. gR10, *AtPDS3* gRNA10. (B) PCR amplification of the DNA of randomly selected albino seedlings from three T2 populations of vCASΦ and nCASΦ U6::*AtPDS3* gR10 in the *rdr6-15* background for a fragment of the CasΦ-2 transgene.

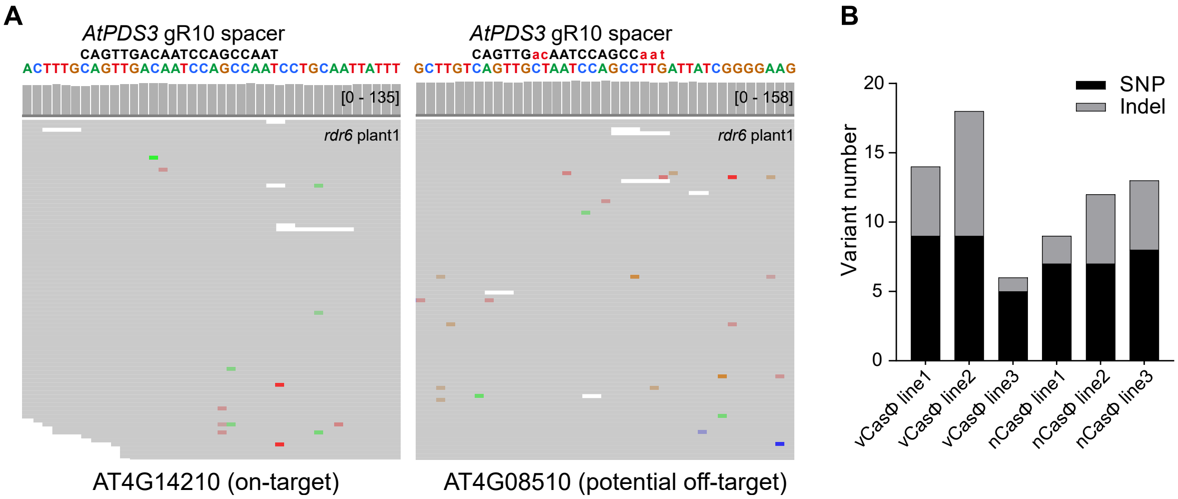

Fig. S8. Summary of high confidence variants discovered in the transgene-free albino T2 seedlings. (A) Screenshots of aligned reads and coverage of a control *rdr6-15* plant at the *AtPDS3* (*AT4G14210*) gRNA10 target region (left) and a potential off-target site (*AT4G08510*) (right). Capitalized and colored sequences are the reference genomic sequences at these two loci. *AtPDS3* gRNA10 spacer sequence is shown in black letters with uncapitalized red letters showing the mismatched nucleotides between *AtPDS3* gRNA10 spacer and the potential off-target site. (B) The number of high confidence SNPs and Indels identified genome wide for the sequenced transgene-free albino seedlings.

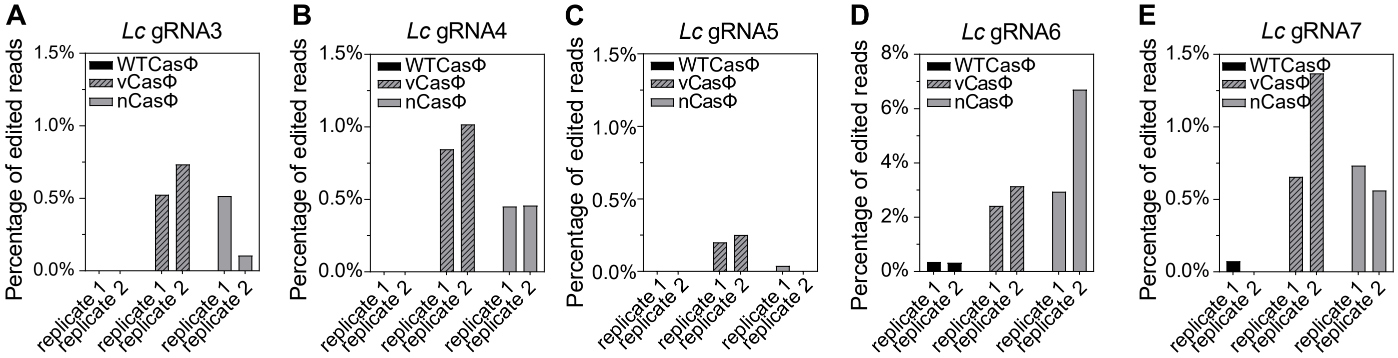

Fig. S9. The editing of *Lc* gene by CasΦ in maize protoplasts. (A)-(E) RNPs reconstituted with WTCasΦ, vCasΦ and nCasΦ proteins and 11 guide RNAs targeting the maize *Lc* gene were transfected into maize protoplasts. Editing of the target region was detected with five out of the 11 guide RNAs tested and editing efficiencies of the two replicate transfections are shown. (A) *Lc* gRNA3. (B) *Lc* gRNA4. (C) *Lc* gRNA5. (D) *Lc* gRNA6. (E) *Lc* gRNA7.

Table S1. Detailed editing events detected in version 1 construct with *AtPDS3* gRNA10 in T1 plant # 33.

tissue	editing event*	supporting reads	total edited reads	total reads	editing efficiency (%)
flower a	-5:6D	284	284	3383814	0.0083929
leaf a	-5:6D	1656570	1660178	3671170	45.22204093
	-4:6D	2728			
	-6:6D	657			
	-1:5D	114			
	-2:6D	109			
leaf b	-5:6D	1922514	1926043	3887866	49.5398504
	-4:6D	2701			
	-6:6D	697			
	-2:6D	131			
leaf c	-5:6D	425	529	3616680	0.01462667
	-4:7D	104			
leaf d	-5:6D	425481	426469	3954723	10.78378941
	-4:6D	882			
	-6:6D	106			

*Editing events are shown as: (position where the editing starts) : (number of nucleotides of) D (deletion) or I (insertion). position 0 is between the 18th and 19th nucleotides of the guide, so that the 18th nucleotide is position -1, the 19th nucleotide is position +1.

Table S2. Detailed number of total seedlings and albino seedlings scored in T2 populations of WTCasΦ U6::gR10, vCasΦ U6::gR10 and nCasΦ U6::gR10 in the *rdr6* background.

rdr6 WTCasΦ U6::gR10			*rdr6* vCasΦ U6::gR10			*rdr6* nCasΦ U6::gR10		
1 out of 31 screened T2 populations have albino seedlings			6 out of 30 screened T2 populations have albino seedlings			14 out of 29 screened T2 populations have albino seedlings		
population number	albino/total seedling number	% of albino seedlings	population number	albino/total seedling number	% of albino seedlings	population number	albino/total seedling number	% of albino seedlings
T2-17	1/313	0.32%	T2-13	18/294	6.12%	T2-10	13/214	6.07%
T2-1	0/317	0%	T2-26	4/239	1.67%	T2-8	3/50	6.00%
T2-2	0/319	0%	T2-29	5/334	1.50%	T2-2	8/150	5.33%
T2-3	0/321	0%	T2-16	2/229	0.87%	T2-6	7/191	3.66%
T2-4	0/334	0%	T2-25	2/332	0.60%	T2-16	1/37	2.70%
T2-5	0/285	0%	T2-30	1/361	0.28%	T2-25	2/100	2.00%
T2-6	0/285	0%	T2-1	0/207	0%	T2-26	2/100	2.00%
T2-7	0/198	0%	T2-2	0/214	0%	T2-20	4/203	1.97%
T2-8	0/352	0%	T2-3	0/201	0%	T2-9	3/193	1.55%
T2-9	0/339	0%	T2-4	0/192	0%	T2-3	3/201	1.49%
T2-10	0/148	0%	T2-5	0/211	0%	T2-21	2/145	1.38%
T2-11	0/256	0%	T2-6	0/191	0%	T2-17	2/175	1.14%
T2-12	0/134	0%	T2-7	0/231	0%	T2-12	1/170	0.59%
T2-13	0/285	0%	T2-8	0/225	0%	T2-4	1/180	0.56%
T2-14	0/327	0%	T2-9	0/240	0%	T2-1	0/87	0%
T2-15	0/323	0%	T2-10	0/235	0%	T2-5	0/164	0%
T2-16	0/286	0%	T2-11	0/351	0%	T2-7	0/106	0%
T2-18	0/297	0%	T2-12	0/271	0%	T2-11	0/197	0%
T2-19	0/339	0%	T2-14	0/230	0%	T2-13	0/150	0%
T2-20	0/291	0%	T2-15	0/345	0%	T2-14	0/180	0%
T2-21	0/193	0%	T2-17	0/341	0%	T2-15	0/200	0%
T2-22	0/271	0%	T2-18	0/300	0%	T2-18	0/200	0%
T2-23	0/342	0%	T2-19	0/276	0%	T2-19	0/87	0%
T2-24	0/378	0%	T2-20	0/314	0%	T2-22	0/100	0%
T2-25	0/286	0%	T2-21	0/300	0%	T2-23	0/100	0%
T2-26	0/336	0%	T2-22	0/239	0%	T2-24	0/100	0%
T2-27	0/242	0%	T2-23	0/295	0%	T2-27	0/100	0%
T2-28	0/311	0%	T2-24	0/368	0%	T2-28	0/96	0%
T2-29	0/289	0%	T2-27	0/350	0%	T2-29	0/93	0%
T2-30	0/295	0%	T2-28	0/216	0%			
T2-31	0/188	0%						

Table S3. Summary of the analysis of whole genome sequencing data of the *rdr6-15* control and CasΦ transgene-free T2 albino seedlings.

		rdr6 control				T2 transgene-negative plant					
						rdr6 vCasphi U6PDS3gR10			*rdr6 nCasphi U6PDS3gR10*		
		plant 1	plant 2	plant 3	plant 4	line1	line2	line3	line1	line2	line3
Reads Number		104272905	244547548	57313755	46421199	150763760	87663735	136018868	117908027	126570163	154534923
Reads Length		150	150	150	150	150	150	150	150	150	150
Reads Type		PE	PE	PE	PE	PE	PE	PE	PE	PE	PE
Coverage		261.41	613.07	143.68	116.38	377.96	219.77	340.99	295.59	317.30	387.41
Mapped reads		102752061	241841799	56528541	45991324	148973332	86547235	134468420	116178427	125262252	152400339
Reads Mapping Rate		98.5415%	98.8936%	98.6300%	99.0740%	98.8124%	98.7264%	98.8601%	98.5331%	98.9667%	98.6187%
Duplication Rate		18.76%	20.72%	10.98%	9.22%	22.70%	17.06%	19.49%	17.93%	20.99%	19.87%
Covered Genome		99.8307%	99.8285%	99.8295%	99.8285%	99.8328%	99.8312%	99.8321%	99.8319%	99.8320%	99.8319%
GATK	SNP	9229	9169	9490	9364	9620	9403	9233	9367	9264	9080
	InDel	7669	6787	8338	7123	8520	9170	8606	8399	9500	7914
Strelka 2	SNP	3419	3840	2983	2725	3726	3374	4033	3841	3713	4610
	InDel	5880	5461	5656	4690	9526	6367	6795	6436	10063	6133
GATK and Strelka2 overlapping SNP+InDel		NA	NA	NA	NA	9083	7547	8046	7450	9727	7938
Filter *rdr6* background		NA	NA	NA	NA	504	304	239	197	666	149
Filter depth < 30		NA	NA	NA	NA	491	293	226	187	649	135
Filter Reference/Alternate allele reads ratio > 3		NA	NA	NA	NA	203	162	124	94	293	66
Overlap with Cas-OFFinder predicted off-targets		NA	NA	NA	NA	0	0	0	0	0	0

Table S4. High confidence variants (excluding the *AtPDS3* gRNA10 site) in CasΦ transgene-free T2 albino seedlings.

Chromosome	Position	Reference sequence	Variant sequence	T2 transgene-negative plant					
				rdr6 vCasphi U6PDS3gR10			*rdr6 nCasphi U6PDS3gR10*		
				line1	line2	line3	line1	line2	line3
chr1	6785692	T	TC	ND*	Y*	ND	ND	ND	ND
chr1	7071325	G	A	ND	ND	Y	ND	ND	ND
chr1	7636794	G	A	ND	Y	ND	ND	ND	ND
chr1	9937635	GTTGTA	G	ND	ND	ND	ND	Y	ND
chr1	11229308	GTCTTTGTGTGAGC	G	ND	Y	ND	ND	ND	ND
chr1	11510229	C	T	ND	ND	ND	ND	ND	Y
chr1	12571948	A	T	ND	ND	Y	ND	ND	ND
chr1	13829012	A	T	ND	ND	ND	ND	ND	Y
chr1	19941306	TGCAATGAGGTTTTG	T	Y	ND	ND	ND	ND	ND
chr1	22302430	GAAAGAAAC	G	ND	ND	ND	ND	ND	Y
chr1	26337186	ATC	A	ND	ND	ND	Y	ND	ND
chr1	27602641	A	T	Y	ND	ND	ND	ND	ND
chr1	28528239	C	T	ND	ND	ND	Y	ND	ND
chr1	29180720	CT	C	ND	ND	ND	ND	ND	Y
chr2	1123998	G	A	ND	Y	ND	ND	ND	ND
chr2	1775712	G	A	Y	ND	ND	ND	ND	ND
chr2	2396463	A	G	ND	ND	ND	ND	ND	Y
chr2	3037107	T	A	ND	Y	ND	ND	ND	ND
chr2	3189614	T	A	Y	ND	ND	ND	ND	ND
chr2	3778595	GA	G	ND	Y	ND	ND	ND	ND
chr2	4070245	G	T	ND	ND	ND	ND	Y	ND
chr2	4473538	G	A	ND	ND	ND	ND	Y	ND
chr2	4597486	T	C	ND	ND	ND	Y	ND	ND
chr2	5939955	A	T	ND	ND	ND	ND	ND	Y
chr2	9981681	TTGATCAAGTAAATG ACATA	T	ND	ND	ND	ND	Y	ND
chr2	9982805	TA	T	ND	ND	Y	ND	ND	ND
chr2	12499196	T	TTA	ND	Y	ND	ND	ND	ND
chr2	13166249	CAT	C	ND	Y	ND	ND	ND	ND
chr2	15371283	TGAAG	T	Y	ND	ND	ND	ND	ND
chr2	15371288	TCTAAATA	T	Y	ND	ND	ND	ND	ND
chr2	15371297	TAA	T	Y	ND	ND	ND	ND	ND
chr2	15371300	C	T	Y	ND	ND	ND	ND	ND
chr2	15371303	A	T	Y	ND	ND	ND	ND	ND
chr2	15371304	C	G	Y	ND	ND	ND	ND	ND
chr2	19035384	T	C	ND	ND	ND	ND	ND	Y
chr2	19623291	C	T	ND	ND	Y	ND	ND	ND
chr3	2788054	A	T	Y	ND	ND	ND	ND	ND
chr3	4305186	G	T	ND	ND	Y	ND	ND	ND
chr3	5085451	TAGGGTCTA	T	ND	ND	ND	ND	Y	ND

chr3	8206493	G	A	ND	Y	ND	ND	ND	ND
chr3	8609529	C	T	ND	ND	ND	ND	Y	ND
chr3	12512717	C	T	ND	ND	ND	Y	ND	ND
chr3	12613340	A	T	ND	Y	ND	ND	ND	ND
chr3	14064862	C	T	ND	ND	ND	Y	ND	ND
chr3	15021799	A	T	ND	ND	ND	ND	Y	ND
chr3	16056216	C	G	ND	ND	ND	ND	Y	ND
chr3	16378772	TATACCTATACGA	T	ND	ND	ND	Y	ND	ND
chr3	16717520	TGACGAGCTTGAG	T	ND	Y	ND	ND	ND	ND
chr3	17108050	G	A	ND	ND	ND	Y	ND	ND
chr4	1843939	G	A	ND	ND	ND	ND	ND	Y
chr4	2261579	T	C	ND	ND	ND	ND	Y	ND
chr4	3360240	C	T	Y	ND	ND	ND	ND	ND
chr4	5305862	G	A	Y	ND	ND	ND	ND	ND
chr4	6581636	C	CA	Y	ND	ND	ND	ND	ND
chr4	8633317	TG	T	ND	ND	ND	ND	ND	Y
chr4	8801901	G	A	ND	ND	ND	ND	ND	Y
chr4	10685528	C	CA	ND	Y	ND	ND	ND	ND
chr4	15242376	T	C	ND	Y	ND	ND	ND	ND
chr5	1309571	A	G	ND	ND	ND	ND	ND	Y
chr5	2561456	ATATGGTTTTGTTAACCGTG	A	ND	ND	ND	ND	Y	ND
chr5	6762332	AAGTTTG	A	ND	ND	ND	ND	ND	Y
chr5	8524857	T	A	ND	ND	ND	ND	Y	ND
chr5	9697518	CCGTCAAAAACTATA	C	ND	Y	ND	ND	ND	ND
chr5	10036901	G	A	ND	ND	ND	Y	ND	ND
chr5	10444172	C	T	ND	Y	ND	ND	ND	ND
chr5	11347449	A	T	ND	Y	Y	ND	ND	ND
chr5	12750772	G	A	ND	ND	ND	Y	ND	ND
chr5	13934573	GA	G	ND	ND	ND	ND	Y	ND
chr5	21062569	C	T	ND	Y	ND	ND	ND	ND
chr5	23985255	TA	T	ND	ND	ND	ND	ND	Y
chr5	26879986	TTTAC	T	ND	Y	ND	ND	ND	ND

* ND, not detected; Y, detected.

Table S5. Sequence of guide RNAs used.

Purpose	CasΦ Guide RNA repeat sequence (common to all guides and on 5' of spacer sequence)		
For plasmid vectors	GTCGGAACGCTCAACGATTGCCCCTCACGAGGGGAC		
For RNPs	CAACGATTGCCCCTCACGAGGGGAC		
**Guide RNA name**	**Guide RNA spacer sequence (Denoted in DNA sequence)**	**PAM**	**Direction relative to target gene**
AtPDS3 gR8	TTGTTCCGCAAAATAGCCCA	TCG	reverse
AtPDS3 gR10 (20bp)	CAGTTGACAATCCAGCCAAT	TTG	reverse
AtPDS3 gR10 (30bp)	CAGTTGACAATCCAGCCAATCCTGCAATTA	TTG	reverse
scramble control	GCGACACGACUCAUUAUA	none	not applicable
FWA gR1	TCCCATTCAACATTCATACG	TTA	forward
FWA gR2	TCGAAGCCCATACATCTTTC	TTA	forward
FWA gR3	TGGGCCGAAGCCCATACATC	TTA	forward
FWA gR4	TGGTTCTATACTAATATCAA	TTA	forward
FWA gR5	ATATTAGTATAGAACCATAA	TTG	reverse
FWA gR6	GTATAGAACCATAACAAAAG	TTA	reverse
FWA gR7	CTAAATTTAGTAAAGAATCA	TTA	forward
FWA gR8	GTAATCAATGGTTATTGTGA	TTA	reverse
FWA gR9	TGAAATGAAATTTAACTTTT	TTG	reverse
FWA gR10	GTTATCTAAATAAAACTAGG	TTA	forward
Lc gR1	TGGACAGAGCTCCAAGTGAC	TTA	reverse
Lc gR2	CTCGGTCACTTGGAGCTCTG	TTG	forward
Lc gR3	GAGCTCTGTCCATAAATTAA	TTG	forward
Lc gR4	TTGCCAACATAGAGTGTACG	TTA	forward
Lc gR5	CCAACATAGAGTGTACGTGG	TTG	forward
Lc gR6	CAGAAGCTAAACTCAACCAG	TTA	forward
Lc gR7	GCTTCTGTAACACTACTGCT	TTA	reverse
Lc gR8	TCTTTGGTGGAGCTCTGGTT	TTG	reverse
Lc gR9	CTTGCAAATTGCATGCACGA	TTA	forward
Lc gR10	CAAATTGCATGCACGAGCTA	TTG	forward
Lc gR11	CATGCACGAGCTAGAATTAT	TTG	forward

Table S6. Plasmids generated in this study.

Plasmid name	Detailed information
HBT_pcoCASphi_version1	cloning vector for sequence of FLAG-SV40NLS-CASphi-withIV2intron-nucleoplasminNLS (Version 1 arrangment)
HBT_pcoCASphi_version2	cloning vector for sequence of CASphi-withIV2intron-2xSV40NLS-2xFLAG (Version 2 arrangment)
pC1300_pUB10_pcoCASphi_E9t_MCS_version1	Binary vector with Arabidopsis codon optimized Casphi driven by UBQ10 promoter and RbcsE9 terminator. Design of NLS and Flag tag is indicated in Figure 1a version 1 plasmid.
pC1300_pUB10_pcoCASphi_E9t_MCS_version2	Binary vector with Arabidopsis codon optimized Casphi driven by UBQ10 promoter and RbcsE9 terminator. Design of NLS and Flag tag is indicated in Figure 1a version 2 plasmid.
pC1300_pUB10_pcoCASphi_E9t_version1_U6_AtPDS3_gRNA10	AtPDS3 guide RNA10 driven by AtU6-26 promoter was cloned into pC1300_pUB10_pcoCASphi_E9t_MCS_version1 plasmid
pC1300_pUB10_pcoCASphi_E9t_version2_U6_AtPDS3_gRNA10	AtPDS3 guide RNA10 driven by AtU6-26 promoter was cloned into pC1300_pUB10_pcoCASphi_E9t_MCS_version2 plasmid
pC1300_pUB10_pcoCASphi_E9t_V2_CmYLCVp_35sT_A_form_AtPDS3_gRNA10	single AtPDS3 gRNA10 driven by CmYLCV promoter and 35S terminator
pC1300_pUB10_pcoCASphi_E9t_V2_CmYLCVp_35sT_B_form_AtPDS3_gRNA10	single AtPDS3 gRNA10 with an extra Casphi repeat sequence at the 3' end driven by CmYLCV promoter and 35S terminator
pC1300_pUB10_pcoCASphi_E9t_V2_CmYLCVp_35sT_C_form_AtPDS3_gRNA10	triple AtPDS3 gRNA10 array driven by CmYLCV promoter and 35S termnator
pC1300_pUB10_pcoCASphi_E9t_V2_2x35Sp_HSP18t_A_form_AtPDS3_gRNA10	single AtPDS3 gRNA10 driven by 2x35S promoter and HSP18 terminator
pC1300_pUB10_pcoCASphi_E9t_V2_2x35Sp_HSP18t_B_form_AtPDS3_gRNA10	single AtPDS3 gRNA10 with an extra Casphi repeat sequence at the 3' end driven by 2x35S promoter and HSP18 terminator
pC1300_pUB10_pcoCASphi_E9t_V2_2x35Sp_HSP18t_C_form_AtPDS3_gRNA10	triple AtPDS3 gRNA10 driven by 2x35S promoter and HSP18 terminator
pC1300_pUB10_pcoCASphi_E9t_V2_pUB10_E9t_A_form_AtPDS3_gRNA10	single AtPDS3 gRNA10 driven by UBQ10 promoter and Rbcs E9 terminator
pC1300_pUB10_pcoCASphi_E9t_V2_pUB10_E9t_B_form_AtPDS3_gRNA10	single AtPDS3 gRNA10 with an extra Casphi repeat sequence at the 3' end driven by UBQ10 promoter and Rbcs E9 terminator
pC1300_pUB10_pcoCASphi_E9t_V2_pUB10_E9t_C_form_AtPDS3_gRNA10	triple AtPDS3 gRNA10 driven by UBQ10 promoter and Rbcs E9 terminator
pC1300_pUB10_pcoCASphi_E9t_V2_CmYLCVp_35sT_A_form_AtPDS3_gRNA10_30bp_spacer	single AtPDS3 gRNA10 with 30bp spacer sequence driven by CmYLCV promoter and 35S terminator
pC1300_pUB10_pcoCASphi_E9t_V2_CmYLCVp_35sT_C_form_AtPDS3_gRNA10_30bp_spacer	triple AtPDS3 gRNA10 with 30bp spacer sequence array driven by CmYLCV promoter and 35S termnator
pC1300_pUB10_pcoCASphi_E9t_V2_2x35Sp_HSP18t_C_form_AtPDS3_gRNA10_30bp_spacer	triple AtPDS3 gRNA10 with 30bp spacer sequence driven by 2x35S promoter and HSP18 terminator
pC1300_pUB10_pcoCASphi_E9t_V2_pUB10_E9t_C_form_AtPDS3_gRNA10_30bp_spacer	triple AtPDS3 gRNA10 with 30bp spacer sequence driven by UBQ10 promoter and Rbcs E9 terminator
pC1300_pUB10_pcoCASphi_E9t_V2_CmYLCVp_35sT_ribozyme_AtPDS3_gRNA10	single AtPDS3 gRNA10 flanked by ribozymes driven by CmYLCV promoter and 35S terminator
pC1300_pUB10_pcoCASphi_E9t_V2_2x35Sp_HSP18t_ribozyme_AtPDS3_gRNA10	single AtPDS3 gRNA10 flanked by ribozymes driven by 2x35S promoter and HSP18 terminator
pC1300_pUB10_pcoCASphi_E9t_V2_pUB10_E9t_ribozyme_AtPDS3_gRNA10	single AtPDS3 gRNA10 flanked by ribozymes driven by UBQ10 promoter and Rbcs E9 terminator

pC1300_pUB10_pco-vCASphi_E9t_MCS_version2	Binary vector with Arabidopsis codon optimized vCasphi driven by UBQ10 promoter and RbcsE9 terminator. Design of NLS and Flag tag is same to the pC1300_pUB10_pcoCASphi_E9t_MCS_version2 plasmid.
pC1300_pUB10_pco-nCASphi_E9t_MCS_version2	Binary vector with Arabidopsis codon optimized nCasphi driven by UBQ10 promoter and RbcsE9 terminator. Design of NLS and Flag tag is same to the pC1300_pUB10_pcoCASphi_E9t_MCS_version2 plasmid.
pC1300_pUB10_pcoCASphi_E9t_V2_U6_AtPDS3_gRNA8	AtPDS3 guide RNA8 driven by AtU6-26 promoter was cloned into pC1300_pUB10_pcoCASphi_E9t_MCS_version2 plasmid
pC1300_pUB10_pcoCASphi_E9t_V2_U6_FWA_gRNA1	FWA guide RNA1 driven by AtU6-26 promoter was cloned into pC1300_pUB10_pcoCASphi_E9t_MCS_version2 plasmid
pC1300_pUB10_pcoCASphi_E9t_V2_U6_FWA_gRNA4	FWA guide RNA4 driven by AtU6-26 promoter was cloned into pC1300_pUB10_pcoCASphi_E9t_MCS_version2 plasmid
pC1300_pUB10_pcoCASphi_E9t_V2_U6_FWA_gRNA5	FWA guide RNA5 driven by AtU6-26 promoter was cloned into pC1300_pUB10_pcoCASphi_E9t_MCS_version2 plasmid
pC1300_pUB10_pcoCASphi_E9t_V2_U6_FWA_gRNA6	FWA guide RNA6 driven by AtU6-26 promoter was cloned into pC1300_pUB10_pcoCASphi_E9t_MCS_version2 plasmid
pC1300_pUB10_pco-vCASphi_E9t_V2_U6_AtPDS3_gRNA8	AtPDS3 guide RNA8 driven by AtU6-26 promoter was cloned into pC1300_pUB10_pco-vCASphi_E9t_MCS_version2 plasmid
pC1300_pUB10_pco-vCASphi_E9t_V2_U6_AtPDS3_gRNA10	AtPDS3 guide RNA10 driven by AtU6-26 promoter was cloned into pC1300_pUB10_pco-vCASphi_E9t_MCS_version2 plasmid
pC1300_pUB10_pco-vCASphi_E9t_V2_U6_FWA_gRNA1	FWA guide RNA1 driven by AtU6-26 promoter was cloned into pC1300_pUB10_pco-vCASphi_E9t_MCS_version2 plasmid
pC1300_pUB10_pco-vCASphi_E9t_V2_U6_FWA_gRNA4	FWA guide RNA4 driven by AtU6-26 promoter was cloned into pC1300_pUB10_pco-vCASphi_E9t_MCS_version2 plasmid
pC1300_pUB10_pco-vCASphi_E9t_V2_U6_FWA_gRNA5	FWA guide RNA5 driven by AtU6-26 promoter was cloned into pC1300_pUB10_pco-vCASphi_E9t_MCS_version2 plasmid
pC1300_pUB10_pco-vCASphi_E9t_V2_U6_FWA_gRNA6	FWA guide RNA6 driven by AtU6-26 promoter was cloned into pC1300_pUB10_pco-vCASphi_E9t_MCS_version2 plasmid
pC1300_pUB10_pco-nCASphi_E9t_V2_U6_AtPDS3_gRNA8	AtPDS3 guide RNA8 driven by AtU6-26 promoter was cloned into pC1300_pUB10_pco-nCASphi_E9t_MCS_version2 plasmid
pC1300_pUB10_pco-nCASphi_E9t_V2_U6_AtPDS3_gRNA10	AtPDS3 guide RNA10 driven by AtU6-26 promoter was cloned into pC1300_pUB10_pco-nCASphi_E9t_MCS_version2 plasmid
pC1300_pUB10_pco-nCASphi_E9t_V2_U6_FWA_gRNA1	FWA guide RNA1 driven by AtU6-26 promoter was cloned into pC1300_pUB10_pco-nCASphi_E9t_MCS_version2 plasmid
pC1300_pUB10_pco-nCASphi_E9t_V2_U6_FWA_gRNA4	FWA guide RNA4 driven by AtU6-26 promoter was cloned into pC1300_pUB10_pco-nCASphi_E9t_MCS_version2 plasmid
pC1300_pUB10_pco-nCASphi_E9t_V2_U6_FWA_gRNA5	FWA guide RNA5 driven by AtU6-26 promoter was cloned into pC1300_pUB10_pco-nCASphi_E9t_MCS_version2 plasmid
pC1300_pUB10_pco-nCASphi_E9t_V2_U6_FWA_gRNA6	FWA guide RNA6 driven by AtU6-26 promoter was cloned into pC1300_pUB10_pco-nCASphi_E9t_MCS_version2 plasmid

pC1300_pUB10_pco-vCASphi_E9t_V2_CmYLCVp_35sT_ribozyme_AtPDS3_gRNA10	the cassette of single AtPDS3 guide RNA10 flanded by ribozymes driven by the CmYLCV promoter and 35S terminator was cloned into the pC1300_pUB10_pco-vCASphi_E9t_MCS_version2 vector
pC1300_pUB10_pco-vCASphi_E9t_V2_pUB10_E9t_ribozyme_AtPDS3_gRNA10	the cassette of single AtPDS3 guide RNA10 flanded by ribozymes driven by the UBQ10 promoter and Rbcs E9 terminator was cloned into the pC1300_pUB10_pco-vCASphi_E9t_MCS_version2 vector
pC1300_pUB10_pco-nCASphi_E9t_V2_CmYLCVp_35sT_ribozyme_AtPDS3_gRNA10	the cassette of single AtPDS3 guide RNA10 flanded by ribozymes driven by the CmYLCV promoter and 35S terminator was cloned into the pC1300_pUB10_pco-nCASphi_E9t_MCS_version2 vector
pC1300_pUB10_pco-nCASphi_E9t_V2_pUB10_E9t_ribozyme_AtPDS3_gRNA10	the cassette of single AtPDS3 guide RNA10 flanded by ribozymes driven by the UBQ10 promoter and Rbcs E9 terminator was cloned into the pC1300_pUB10_pco-nCASphi_E9t_MCS_version2 vector

Table S7. Sequence of primers and synthesized double stranded DNA.

For amplicon sequencing:		
**Oligo name**	**Oligo sequence**	**Purpose and details**
20685	ACACTCTTTCCCTACACGACGCTCTTCCGATCTCATGGCTGGCAAAAGTCCAATAGCA	AtPDS3 gR8 amplicon step1 FW
20686	GTGACTGGAGTTCAGACGTGTGCTCTTCCGATCTACTGGTCAAGGCAAGACGATATAACT	AtPDS3 gR8 amplicon step1 RV
20681	ACACTCTTTCCCTACACGACGCTCTTCCGATCTGTACCTTTCCACCAAGAACATCTCT	AtPDS3 gR10 amplicon step1 FW
20682	GTGACTGGAGTTCAGACGTGTGCTCTTCCGATCTTCCTCGTCCTGCTAAGCCTTTGA	AtPDS3 gR10 amplicon step1 RV
21405	ACACTCTTTCCCTACACGACGCTCTTCCGATCTTTCGTTCTTGTGTCATGTAATAGATTACT	FWA gR10 amplicon step1 FW
21406	GTGACTGGAGTTCAGACGTGTGCTCTTCCGATCTGCCCATAACTCTTTGATATTAGTATAGA	FWA gR10 amplicon step1 RV
21407	ACACTCTTTCCCTACACGACGCTCTTCCGATCTAGGCCATCCATGGATGGTTTCA	FWA gR7, gR8, gR9 amplicon step1 FW
21408	GTGACTGGAGTTCAGACGTGTGCTCTTCCGATCTGAATATATGAGATTCTCGACGGAAAGA	FWA gR7, gR8, gR9 amplicon step1 RV
21409	ACACTCTTTCCCTACACGACGCTCTTCCGATCTGAATATATGAGATTCTCGACGGAAAGA	FWA gR4, gR5, gR6 amplicon step1 FW
21410	GTGACTGGAGTTCAGACGTGTGCTCTTCCGATCTAGGCCATCCATGGATGGTTTCA	FWA gR4, gR5, gR6 amplicon step1 RV
21411	ACACTCTTTCCCTACACGACGCTCTTCCGATCTCGGAAAGATGTATGGGCTTCGAT	FWA gR3 amplicom step1 C12J FW, pair with primer 21410 for PCR reaction
21412	ACACTCTTTCCCTACACGACGCTCTTCCGATCTATAGCACTTGGACCAATGGCGAA	FWA gR1 amplicon step1 FW, pair with primer 21414 for PCR reaction
21413	ACACTCTTTCCCTACACGACGCTCTTCCGATCTCGTATGAATGTTGAATGGGATAAGGT	FWA gR2 amplicon step1 FW, pair with primer 21414 for PCR reaction
21414	GTGACTGGAGTTCAGACGTGTGCTCTTCCGATCTAGAATCAATTGGGTTTAGTGTTTACTTGT	FWA gR1, gR2 amplicon step1 RV
23997	ACACTCTTTCCCTACACGACGCTCTTCCGATCTGACGGGGTAGATGCCTACAGA	casphi lc gR1,gR2,gR3 amplicon step1 FW
23998	GTGACTGGAGTTCAGACGTGTGCTCTTCCGATCTTATAGCGTCAGAGAACTTAGATCTGA	casphi lc gR1,gR2,gR3 ,gR6,gR7,gR8 amplicon step1 RV
23999	ACACTCTTTCCCTACACGACGCTCTTCCGATCTAGTGACCGAGCAAGACGGTGA	casphi lc gR6,gR7,gR8 amplicon step1 FW
24000	ACACTCTTTCCCTACACGACGCTCTTCCGATCTGCTCCTCACTAGCTACCAAGAG	casphi lc gR4,gR5 amplicon step1 FW
ZL21	GTGACTGGAGTTCAGACGTGTGCTCTTCCGATCTCTACTGATCATCAGACGATGCCT	casphi lc gR4,gR5 amplicon step1 RV
ZL22	ACACTCTTTCCCTACACGACGCTCTTCCGATCTCACACAGTATCAGCTGGCACA	casphi lc gR9,gR10,gR11 amplicon step1 FW
ZL23	GTGACTGGAGTTCAGACGTGCTCTTCCGATCTCTGGTTGAGTGTCTGAAATGGAC	casphi lc gR9,gR10,gR11 amplicon step1 RV

**For cloning and genotyping:**		
**Oligo name**	**Oligo sequence**	**Purpose and details**
20478	AGCAGCTGGAACTCCGTGGA	FW primer to amplify HBT backbone for HBT_pcoCASphi version1
20479	AAGAGACCAGCTGCTACCAAGA	RV primer to amplify HBT backbone for HBT_pcoCASphi version1
20497	GGATCCACGGAGCAAGGGGA	FW primer to amplify HBT backbone for HBT_pcoCASphi version2
20498	TGACTGCAGATCGTTCAAACATTTGGCA	RV primer to amplify HBT backbone for HBT_pcoCASphi version2
20501	TCCACGGAGTTCCAGCTGCTATGCCGAAGCCCGCCG TCGA	FW primer to amplify pcoCASphi 1st fragment for in-fusion reaction to generate HBT_pcoCASphi_version1
20507	CTTGCTCCGTGGATCCATGCCGA	FW primer to amplify pcoCASphi 1st fragment for in-fusion reaction to generate HBT_pcoCASphi_version2
20502	CTGGCGTTTCTTAGTAATCCTCTTACGT	RV primer to amplify pcoCASphi 1st fragment for in-fusion reaction to generate HBT_pcoCASphi_version1 and HBT_pcoCASphi_version2
20503	GGATTACTAAGAAACGCCAGGTAAGTTTCTGCTTCT ACCTTTGA	FW primer to amplify IV2 intron for in-fusion reaction to generate HBT_pcoCASphi_version1 and HBT_pcoCASphi_version2
20504	TGGGAGCAATAGTCCTCCACCTGCACATCAACAAAT TTTGGTCA	RV primer to amplify IV2 intron for in-fusion reaction to generate HBT_pcoCASphi_version1 and HBT_pcoCASphi_version2
20505	GTGGAGGACTATTGCTCCCAAGGA	FW primer to amplify pcoCASphi 2nd fragment for in-fusion reaction to generate HBT_pcoCASphi_version1 and HBT_pcoCASphi_version2
20506	TTGGTAGCAGCTGGTCTCTTGGACGTTTGGGACGGC TCTTGA	RV primer to amplify pcoCASphi 2nd fragment for in-fusion reaction to generate HBT_pcoCASphi_version1
20508	GCCAAATGTTTGAACGATCTGCAGTCACT	RV primer to amplify pcoCASphi 2nd fragment for in-fusion reaction to generate HBT_pcoCASphi_version2

20544	ATCACTAGTATCCTAGGAAGGTACCAGTCTAGCTCA ACAGAGCTTTTAACCCA	FW primer to amplify pUBQ10 for in-fusion reaction to generate pC1300_pUB10_pcoCASphi_E9t_MCS_version1 and pC1300_pUB10_pcoCASphi_E9t_MCS_version2 plasmids
20545	TCATCATCCTTGTAATCCATCTGTTAATCAGAAAAAC TCAGATTAATCGA	RV primer to amplify pUBQ10 for in-fusion reaction to generate pC1300_pUB10_pcoCASphi_E9t_MCS_version1
20552	TCGACGGCGGGCTTCGGCATCTGTTAATCAGAAAAA CTCAGATTAATCGA	RV primer to amplify pUBQ10 for in-fusion reaction to generate pC1300_pUB10_pcoCASphi_E9t_MCS_version2
20548	TGAGTTTTTCTGATTAACAGATGGATTACAAGGATG ATGATGATAAGGA	FW primer to amplify 2xFLAG-SV40-Casphi-2-IV2intron-nucleoplasminNLS for in-fusion reaction to generate pC1300_pUB10_pcoCASphi_E9t_MCS_version1
20550	TGAGTTTTTCTGATTAACAGATGCCGAAGCCCGCCG TCGAATCA	FW primer to amplify Casphi-2-IV2intron-2xSV40-2xFLAG for in-fusion reaction to generate pC1300_pUB10_pcoCASphi_E9t_MCS_version2
20549	ATGATACGAACGAAAGCTCTTCACTTCTTCTTCTTAG CCTGTCCA	RV primer to amplify 2xFLAG-SV40-Casphi-2-IV2intron-nucleoplasminNLS for in-fusion reaction to generate pC1300_pUB10_pcoCASphi_E9t_MCS_version1
20551	ATGATACGAACGAAAGCTCTTCACTTGTCGTCGTCA TCCTTATAGT	RV primer to amplify Casphi-2-IV2intron-2xSV40-2xFLAG for in-fusion reaction to generate pC1300_pUB10_pcoCASphi_E9t_MCS_version2
20546	AGGCTAAGAAGAAGAAGTGAAGAGCTTTCGTTCGT ATCATCGGT	FW primer to amplify RbcS E9 terminator for in-fusion reaction to generate pC1300_pUB10_pcoCASphi_E9t_MCS_version1
20553	AGGATGACGACGACAAGTGAAGAGCTTTCGTTCGT ATCATCGGTTTCGA	FW primer to amplify RbcS E9 terminator for in-fusion reaction to generate pC1300_pUB10_pcoCASphi_E9t_MCS_version2

20547	CAGCTATGACCATGATTACGAATTCGTTGTCAATCA ATTGGCAAGTCATAAAATGCA	RV primer to amplify RbcS E9 terminator for in-fusion reaction to generate pC1300_pUB10_pcoCASphi_E9t_MCS_version1 and pC1300_pUB10_pcoCASphi_E9t_MCS_version2 plasmids.
14444	TTCCTAGGATACTAGAAGCTTCGTTGAACAACGGA	FW primer to amplify AtU6-26 gRNA cassette for in-fusion reaction
20665	TGCAGGTCGACTCTAGATCACTAGTGATCAGATGCA GAGAGACT	RV primer to amplify AtU6-26 gRNA cassette for in-fusion reaction
20730	TTGTTCCGCAAAATAGCCCATTTTTTTTGCCATTCTTT TCAAGCTCCA	AtPDS3 gRNA8 FW
20731	TGGGCTATTTTGCGGAACAAGTCCCCTCGTGAGGG GCAATCGTTGA	AtPDS3 gRNA8 RV
20734	CAGTTGACAATCCAGCCAATTTTTTTTTGCCATTCTTT TCAAGCTCCA	AtPDS3 gRNA10 FW
20735	ATTGGCTGGATTGTCAACTGGTCCCCTCGTGAGGGG CAATCGTTGA	AtPDS3 gRNA10 RV
21950	TCCCATTCAACATTCATACGTTTTTTTTGCCATTCTTT TCAAGCTCCATTGTCA	FWA gRNA1 FW
21949	CGTATGAATGTTGAATGGGAGTCCCCTCGTGAGGG GCAATCGTTGAGCGTTCCGA	FWA gRNA1 RV
21952	TGGTTCTATACTAATATCAATTTTTTTTGCCATTCTTT TCAAGCTCCATTGTCA	FWA gRNA4 FW
21951	TTGATATTAGTATAGAACCAGTCCCCTCGTGAGGGG CAATCGTTGAGCGTTCCGA	FWA gRNA4 RV
21954	ATATTAGTATAGAACCATAATTTTTTTTGCCATTCTTT TCAAGCTCCATTGTCA	FWA gRNA5 FW
21953	TTATGGTTCTATACTAATATGTCCCCTCGTGAGGGG CAATCGTTGAGCGTTCCGA	FWA gRNA5 RV
21956	GTATAGAACCATAACAAAAGTTTTTTTTGCCATTCTT TTCAAGCTCCATTGTCA	FWA gRNA6 FW
21955	CTTTTGTTATGGTTCTATACGTCCCCTCGTGAGGGGC AATCGTTGAGCGTTCCGA	FWA gRNA6 RV
21276	GACTGGTACCTTCCTAGGATACTAGTGGCAGACATA CTGTCCCACA	CmYLCV promoter FW
21277	GACTTTGATAGCTTGCTGAGGCAAGCTTAGCTCTTA CCTGTTTTCGT	CmYLCV promoter RV
21278	TTCGATAATTCCTTAATTAACTCGAGTTTCTCCATAA TAATGTGTGA	35S terminator FW
21386	CTGCAGGTCGACTCTAGATCACTAGTTAATTCGGGG GATCTGGATTTTAGTACT	35S terminator RV
21440	CTGTTGAGCTAGACTGGTACCTTCCTAGGATACTAG GCCAGTGCCAAGCTTG	2x35S promoter FW

21388	GACTTTGATAGCTTGCTGAGGCGGGATCCTCTAGAG TCGAGGT	2x35S promoter RV
21389	TTCGATAATTCCTTAATTAAATATGAAGATGAAGAT GAAATATTTGGTGTGT	HSP18.2 terminator FW
21390	CTGCAGGTCGACTCTAGATCACTAGTCTTATCTTTAA TCATATTCCATAGTCCATACCA	HSP18.2 terminator RV
21451	TTGAGCTAGACTGGTACCTTCCTAGGATACTAGAAG TTGTAATGAGTTGCTGGCCTCTCT	TBSinsulator-UBQ10 promoter FW
21392	GACTTTGATAGCTTGCTGAGGCCTGTTAATCAGAAA AACTCAGATTAATCGACA	UBQ10 promoter RV
21393	TTCGATAATTCCTTAATTAAAGAGCTTTCGTTCGTAT CATCGGT	Rbcs E9 terminator FW
21394	CTGCAGGTCGACTCTAGATCACTAGTGTTGTCAATC AATTGGCAAGTCATAAAATGCA	Rbcs E9 terminator RV
single PDS3 gRNA10 FW	GCCTCAGCAAGCTATCAAAGTCGGAACGCTCAACGA TTGCCCCTCACGAGGGGACCAGTT	single PDS3 gRNA10 FW to clone into PolII promoter gRNA cassette
single PDS3 gRNA10 RV	TTAATTAAGGAATTATCGAAATTGGCTGGATTGTCA ACTGGTCCCCTCGTGAGG	single PDS3 gRNA10 RV to clone into PolII promoter gRNA cassette
single PDS3 gR10 + repeat FW	GCCTCAGCAAGCTATCAAAGTCGGAACGCTCAACGA TTGCCCCTCACGAGGGGACCAGTTGACAATCCAGCC AAT	single PDS3 gR10 + repeat FW to clone into PolII promoter gRNA cassette
single PDS3 gR10 + repeat RV	TTAATTAAGGAATTATCGAAGTCCCCTCGTGAGGGG CAATCGTTGAGCGTTCCGACATTGGCTGGATTGTCA ACTG	single PDS3 gR10 + repeat RV to clone into PolII promoter gRNA cassette
triple PDS3 gR10 FW	GCCTCAGCAAGCTATCAAAGTCGGAACGCTCAACGA TTGCCCCTCACGAGGGGACCAGTTGACAATCCAGCC AATGTCGGAACGCTCAACGATTGCCCCTCACGAGG GGACCAGTTGACAATCCAGCCAATGTCGGAACGCTC AACGATTGCCCCTCACGAGGGGACCAGTTGACAATC CAGCCAAT	triple PDS3 gR10 FW to clone into PolII promoter gRNA cassette
triple PDS3 gR10 RV	TTAATTAAGGAATTATCGAAGTCCCCTCGTGAGGGG CAATCGTTGAGCGTTCCGACATTGGCTGGATTGTCA ACTGGTCCCCTCGTGAGGGGCAATCGTTGAGCGTTC CGACATTGGCTGGATTGTCAACTGGTCCCCTCGTGA GGGGCAATCGTTGAGCGTTCCGACATTGGCTGGATT GTCAACTG	triple PDS3 gR10 RV to clone into PolII promoter gRNA cassette
21757	AAGCTTGCCTCAGCAAGCTATCAAAGTCGGAACGCT CAACGATTG	PolII gRNA cassette fw (to clone the gRNA of 30bp spacer into the PolII gRNA cassette)
21760	AACTCGAGTTAATTAAGGAATTATCGAATAATTGCA GGATTGGCTGGATTGTCAACTGGT	PolII gRNA cassette AtPDS3 gRNA10 30bp RV

21763	TGCCTCAGCAAGCTATCAAAGTCGGAACGCTCAACGATTGCCCCTCACGAGGGGACCAGTTGACAATCCAGCCAATCCTGCAATTAGTCGGAACGCTCAACGATTGCCCCTCACGAGGGGACCAGTTGACAATCCAGCCAATCCTGCAATTAGTCGGAACGCTCAACGATTGCCCCTCACGAGGGGACCAGTTGACAATC	30bp spacer PDS3 triple gR10 array fw
21764	AGTTAATTAAGGAATTATCGAAGTCCCCTCGTGAGGGGCAATCGTTGAGCGTTCCGACTAATTGCAGGATTGGCTGGATTGTCAACTGGTCCCCTCGTGAGGGGCAATCGTTGAGCGTTCCGACTAATTGCAGGATTGGCTGGATTGTCAACTGGTCCCCTCGTGAGGGGCAATCGTTGAGCGTTCCGACTAATTGCAGG	30bp spacer PDS3 triple gR10 array rv
21732	TAAGAGCTAAGCTTGCCTCAGCTCCGACCTGATGAGTCCGTGAGGACGAAACGAGTAAGCTCGTCGTCGGAACGCTCAACGATTGCCCCTCACGAGGGGACCAGTTGACAATCCAGCCAATG	PDS3 gR10 + ribozyme fw
21733	TGGAGAAACTCGAGTTAATTAAGTCCCATTCGCCATGCCGAAGCATGTTGCCCAGCCGGCGCCAGCGAGGAGGCTGGGACCATGCCGGCCATTGGCTGGATTGTCAACTGGT	PDS3 gRNA10 + ribozyme rv
21847	TCCTCCTCACCTGAAGATCCGGCCTTCTCATTTCGAAGCTGT	vCasphi mutation RV
21848	GGCCGGATCTTCAGGTGAGGAGGAGGTAGCTACAAATGA	vCasphi mutation FW
21849	TCTTACGTCTATGACTACCCAATCGGT	vCasphi and nCasphi Fragment2 RV
21851	AGCTGCAGCTCGAGCGGCGTTTATTGCAGCCAATCTAGCTCGGGCCTTCTCA	nCasphi mutation RV
21852	GCTGCAATAAACGCCGCTCGAGCTGCAGCTGGATTGCCGGAAATCAAGGCCGAGGA	nCasphi mutation FW
20505	GTGGAGGACTATTGCTCCCAAGGA	Casphi genotyping FW
20639	TCCAGAGCTCTGACCTCTGCT	Casphi genotyping RV
21403	AAGGAGTCATTTTTCACTAAGCATATAGA	FWA fragment amplification FW for in vitro RNP cleavage substrate
21404	CATTTCTAGTGTCTCGACAACGAACA	FWA fragment amplification RV for in vitro RNP cleavage substrate
**For real-time quantitative PCR:**		
**Oligo name**	**Oligo sequence**	**Purpose and details**
11859	GTATGAGTTGCTTCTCCAGCAAAG	IPP2 QPCR FW
11860	GAGGATGGCTGCAACAAGTGT	IPP2 QPCR RV
21056	GGTCGGAACGCTCAACGATTG	CASphi gRNA QPCR FW
21059	ATTGGCTGGATTGTCAACTGGTC	CASphi AtPDS3gR10 QPCR RV
**DNA synthesized:**		

DNA name	sequence	
CASphi-2-2xSV40NLS-2xFLAG	CTTGCTCCGTGGATCCATGCCGAAGCCCGCCGTCGA<br>ATCAGAGTTTTCCAAAGTCCTCAAGAAACACTTTCCT<br>GGGGAGCGTTTTAGGTCTAGCTATATGAAGAGGGG<br>GGGTAAAATTCTGGCAGCACAAGGCGAGGAAGCTG<br>TAGTGGCGTACTTGCAGGGAAAGAGTGAGGAGGA<br>ACCGCCGAATTTTCAGCCGCCGGCGAAGTGCCACGT<br>GGTCACCAAAAGCAGGGATTTCGCAGAATGGCCCA<br>TAATGAAAGCCTCTGAAGCCATACAGAGGTACATCT<br>ATGCGCTCAGCACTACAGAGCGAGCTGCCTGCAAA<br>CCGGGTAAGAGCTCAGAAAGTCACGCGGCCTGGTT<br>CGCGGCTACAGGGGTGAGCAATCACGGCTATTCTC<br>ATGTACAAGGTCTTAACCTGATCTTTGACCACACGCT<br>AGGACGATACGATGGCGTTTTAAAGAAAGTACAGC<br>TTCGAAATGAGAAGGCCCGAGCTAGATTGGAAAGC<br>ATAAACGCCTCACGAGCTGATGAAGGATTGCCGGA<br>AATCAAGGCCGAGGAGGAGGAGGTAGCTACAAAT<br>GAAACAGGTCATCTACTACAGCCGCCAGGCATAAAC<br>CCATCATTCTACGTCTACCAGACCATATCTCCGCAGG<br>CTTACCGACCAAGGGACGAAATAGTGTTACCACCCG<br>AGTACGCCGGTTACGTCAGGGATCCGAACGCTCCG<br>ATTCCACTGGGCGTGGTCAGGAACCGTTGTGACATA<br>CAGAAGGGTTGCCCGGGATATATACCCGAGTGGCA<br>GAGGGAAGCTGGTACGGCAATTAGTCCCAAGACAG<br>GAAAAGCAGTGACGGTTCCAGGACTTAGCCCGAAG<br>AAGAATAAACGTATGCGTAGGTACTGGAGGTCAGA<br>AAAGGAGAAGGCTCAAGATGCACTTCTCGTAACTGT<br>AAGGATAGGTACCGATTGGGTAGTCATAGACGTAA<br>GAGGATTACTAAGAAACGCCAGGTGGAGGACTATT<br>GCTCCCAAGGACATAAGTTTAAATGCACTTCTAGAT<br>TTATTTACCGGTGATCCAGTCATCGATGTCAGACGA<br>AACATCGTGACCTTCACCTATACCTTGGACGCTTGC<br>GGAACTTATGCTAGAAAATGGACTCTCAAGGGAAA<br>ACAGACAAAAGCAACCTTAGATAAACTGACAGCGA<br>CACAAACTGTGGCCTTAGTTGCTATAGATCTGGGAC<br>AAACAAACCCAATTAGCGCGGGTATCAGTCGTGTCA<br>CACAGGAGAACGGGGCCCTCCAGTGCGAACCGCTT<br>GATCGTTTTACATTGCCTGATGACCTTTTGAAAGATA<br>TTTCTGCGTACCGAATTGCATGGGACCGTAACGAGG<br>AGGAACTCAGGGCCAGATCCGTTGAGGCACTCCCA<br>GAGGCACAACAAGCAGAGGTCAGAGCTCTGGACGG<br>GGTCTCCAAAGAGACCGCGCGTACACAGTTGTGCG<br>CGGACTTCGGTCTGGACCCAAAGCGACTACCGTGG<br>GATAAAATGAGTAGCAATACCACGTTCATAAGCGA<br>GGCGCTCCTTTCCAACAGCGTATCCCGTGACCAAGT<br>ATTCTTTACCCCGGCCCCAAAGAAAGGAGCCAAGAA<br>GAAAGCACCGGTGGAAGTGATGCGAAAAGACAGG	

	ACATGGGCGCGAGCGTACAAACCACGACTCTCAGT<br>AGAAGCACAAAAGTTGAAAAATGAGGCTCTTTGGG<br>CTTTGAAGCGTACCTCTCCAGAATATCTAAAGTTGTC<br>ACGACGTAAAGAAGAATTGTGTAGGAGGTCCATTA<br>ATTACGTGATAGAAAAAACTAGAAGGCGAACCCAA<br>TGCCAAATTGTGATCCCCGTTATAGAAGATCTAAAT<br>GTCCGATTTTTCCACGGGTCTGGCAAACGACTCCCG<br>GGCTGGGATAACTTCTTCACGGCAAAGAAGGAAAA<br>CCGATGGTTTATCCAAGGGCTACATAAGGCGTTTTC<br>TGACTTAAGGACCCACCGATCCTTCTACGTGTTCGA<br>GGTGCGTCCAGAGAGAACATCAATCACCTGTCCGA<br>AATGCGGGCACTGTGAAGTGGGGAACAGGGATGG<br>AGAAGCCTTTCAGTGTCTCAGTTGCGGTAAGACATG<br>CAACGCAGATCTGGACGTAGCGACACATAACTTGAC<br>TCAAGTGGCGCTCACCGGCAAAACAATGCCGAAGA<br>GAGAGGAGCCTAGAGATGCACAAGGGACAGCCCCC<br>GCGAGAAAGACGAAGAAAGCTAGCAAGTCAAAGG<br>CACCCCCGGCTGAACGTGAGGATCAGACTCCCGCTC<br>AAGAGCCGTCCCAAACGTCCGGATCCGGACCGAAG<br>AAAAAGCGAAAGGTAGAGGATCCTAAAAAGAAGC<br>GTAAAGTCTCCTTGGGTTCTGGCTCCGACTATAAGG<br>ATGACGATGACAAAGACTATAAGGATGACGACGAC<br>AAGTGACTGCAGATCGTTCAAACATTTGGC	
